# High-Resolution Recording of the Circadian Oscillator in Primary Mouse α- and β-Cell Culture

**DOI:** 10.3389/fendo.2017.00068

**Published:** 2017-04-07

**Authors:** Volodymyr Petrenko, Yvan Gosmain, Charna Dibner

**Affiliations:** ^1^Endocrinology, Diabetes, Hypertension and Nutrition Division, Department of Specialties of Medicine, University Hospital of Geneva, Geneva, Switzerland; ^2^Faculty of Medicine, Department of Cell Physiology and Metabolism, University of Geneva, Geneva, Switzerland; ^3^Institute of Genetics and Genomics in Geneva (iGE3), Geneva, Switzerland; ^4^Diabetes Center of the Faculty of Medicine, University of Geneva, Geneva, Switzerland; ^5^Molecular Diabetes Laboratory, Endocrinology, Diabetes, Hypertension and Nutrition Division, Faculty of Medicine, Department of Specialties of Medicine, University Hospital of Geneva, University of Geneva, Geneva, Switzerland

**Keywords:** mouse pancreatic islet, α- and β-cells, primary culture, *in vitro* synchronization, circadian bioluminescence

## Abstract

Circadian clocks have been developed in evolution as an anticipatory mechanism allowing for adaptation to the constantly changing light environment due to rotation of the Earth. This mechanism is functional in all light-sensitive organisms. There is a considerable body of evidence on the tight connection between the circadian clock and most aspects of physiology and metabolism. Clocks, operative in the pancreatic islets, have caught particular attention in the last years due to recent reports on their critical roles in regulation of insulin secretion and etiology of type 2 diabetes. While β-cell clocks have been extensively studied during the last years, α-cell clocks and their role in islet function and orchestration of glucose metabolism stayed unexplored, largely due to the difficulty to isolate α-cells, which represents a considerable technical challenge. Here, we provide a detailed description of an experimental approach for the isolation of separate mouse α- and β-cell population, culture of isolated primary α- and β-cells, and their subsequent long-term high-resolution circadian bioluminescence recording. For this purpose, a triple reporter *ProGlucagon*-*Venus*/RIP-*Cherry*/*Per2*:*Luciferase* mouse line was established, carrying specific fluorescent reporters for α- and β-cells, and luciferase reporter for monitoring the molecular clockwork. Flow cytometry fluorescence-activated cell sorting allowed separating pure α- and β-cell populations from isolated islets. Experimental conditions, developed by us for the culture of functional primary mouse α- and β-cells for at least 10 days, will be highlighted. Importantly, temporal analysis of freshly isolated α- and β-cells around-the-clock revealed preserved rhythmicity of core clock genes expression. Finally, we describe the setting to assess circadian rhythm in cultured α- and β-cells synchronized *in vitro*. The here-described methodology allows to analyze the functional properties of primary α- and β-cells under physiological or pathophysiological conditions and to assess the islet cellular clock properties.

## Introduction

The circadian system represents a complex anticipatory mechanism developed during evolution in nearly all organisms, allowing to coordinate a plethora of physiological functions to the daily changes of geophysical time. Within this system, a master pacemaker in the hypothalamus orchestrates subsidiary oscillators situated in peripheral organs (1). In fact, myriads of these self-sustained and cell-autonomous oscillators are operative in most cells of the body (2, 3). The molecular composition of central and peripheral oscillators is identical, and it relies on primary and secondary feedback loops of transcription and translation of key core clock components (4). The primary loop comprises the positive limb transcription factors CLOCK and BMAL1, which induce expression of the negative limb elements PERIODS and CRYPTOCHROMES (5). Recent studies provide increasing evidence for a tight connection between the circadian system and metabolism, linking metabolic diseases to circadian misalignments associated with modern life-style, including frequent jetlag, shifted work schedules, and chronic social jetlag (4, 6–10). Studies in clock-deficient genetic rodent models suggest that a number of metabolic defects develop in mice that are deficient for one or two core clock components (11, 12). For instance, *Clock* mutant mice develop hyperphagia, obesity, hyperglycemia, and hypoinsulinemia (12).

There is an increasing evidence for the essential roles of the peripheral circadian clocks operative in endocrine tissues for their transcriptional and functional regulation (13–15). Indeed, most of the hormones, including myokines and adipokines, are secreted in a circadian manner and regulated by respective cell-autonomous oscillators (16, 17). Such cell-autonomous clocks have been recently characterized in pancreatic islets in mice (11, 18) and in humans (18–20). Loss of islet clock function in islet-specific *Bmal1* KO mouse models, either induced during development or in the adult age, resulted in the early onset of type 2 diabetes (T2D) in these mice (11, 18, 21). Moreover, siClock-mediated clock perturbation in adult human islet cells caused disruption in basal and glucose induced insulin secretion by these cells *in vitro* (20). Taken together, these data suggest that circadian oscillators operative in islet cells play an important role in regulating these cell function.

So far, most of the research works were conducted on whole islets, or on insulin secreting β-cells, representing about 80% of total islet cells in mice (22). Therefore, the circadian physiology of glucagon secreting α-cells stayed largely unexplored, due to the difficulty to identify these cells within the complex three dimensional islet structure and to isolate them due to their low abundance (less than 20% of the mouse islet cell population). In an attempt to fill this gap, we hereby report an experimental approach, which allows to (1) efficiently isolate nearly pure populations of mouse α- and β-cells; (2) establish and maintain mouse α- and β-cell primary cultures; (3) study endocrine function of separated α- and β-cells; and (4) assess the circadian properties of primary α- and β-cells, utilizing high-resolution circadian bioluminescence monitoring in living cells synchronized *in vitro*.

## Materials and Methods

### Animal Care and Reporter Mouse Strain

**Figure 1:**
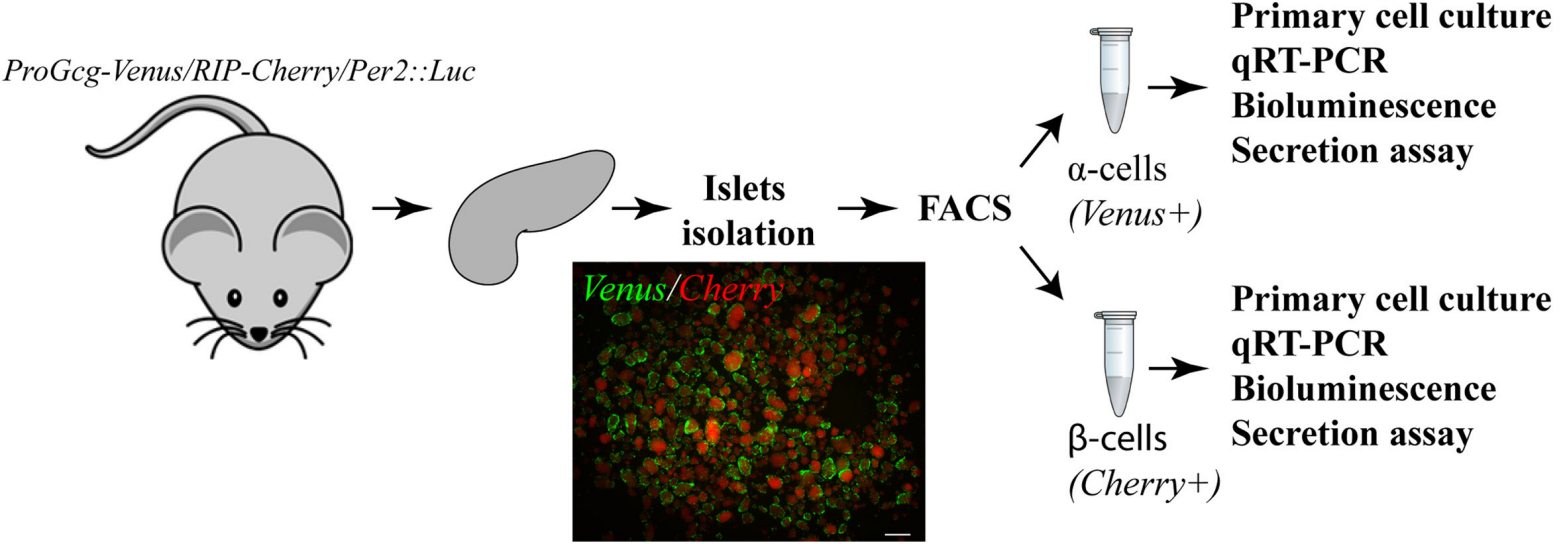
**Overview of the experimental procedures**. Pancreatic islets were isolated from triple transgenic mice (ProGcg-Venus/RIP-Cherry/Per2:Luc). Representative fluorescent image demonstrates pancreatic islets comprising *Venus*-positive α-cells preferentially located on the periphery of islets and *Cherry*-positive β-cells in the core (labeled in green and red, respectively). Following fluorescence-activated cell sorting (FACS) separation, populations of α- and β-cells were subjected to further experimental procedures [primary cultures, quantitative RT-PCR (qRT-PCR) analysis, bioluminescence recording, and assessment of hormone secretion]. Scale bar = 200 μm.

For all experiments a triple reporter mouse strain *ProGlucagon*-*Venus*/RIP-*Cherry*/*Per2*:*Luciferase* (*ProGcg*-*Venus*/RIP-*Cherry*/*Per2*:*Luc*) was derived by crossing the *ProGlucagon*-*Venus(ProGcg-Venus)* reporter mouse (23) with Rat *Insulin2* promoter (RIP)-*Cherry* (RIP-*Cherry*) (24) and *Period2:Luciferase* (*Per2:Luc*) mice (25). *ProGcg-Venus* and *RIP-Cherry* reporters exhibit a high specificity for α- and β-cells, respectively, while the fusion protein PER2:Luciferase, encoded by *Per2:Luc*, is a circadian reporter functionally indistinguishable from the wild-type PER2 protein. The overview of the experimental procedures is illustrated in Figure 1. All experiments were conducted on male mice aged 7–16 weeks under standard animal housing conditions comprising *ad libitum* access to food and water and 12 h light/12 h dark cycles. Islet isolations were performed during morning hours (07:00 a.m.–12:00 a.m.). To study circadian rhythms in freshly isolated α- and β-cells *in vivo*, mice were subjected to night-restricted feeding 2 weeks prior to the experiment and during the entire period of sample collection as described previously (26), with half of the animals entrained with inversed light-dark and feeding cycles during the same period.

### Pancreatic Islet Isolation and Separation of α- and β-Cells

Islets of Langerhans were isolated by a standard procedure based on collagenase (Type XI, Sigma) digestion of pancreas followed by Ficoll purification (27). Briefly, 2 ml of a 2-mg/ml collagenase solution [diluted in Hanks Balanced Salt Solution (HBSS) (Gibco), 4.2 nM sodium bicarbonate, 1 M CaCl_2_, and 1 M HEPES (Gibco)] was injected retrogradely through the ampulla of Vater into the exocrine part of the pancreas, which was dissected and digested in a water bath at 37°C (11–14 min, exact time needs to be calibrated for every new batch of enzyme). The digested mixture was then washed in HBSS, supplemented with 0.3% free fatty acid bovine serum albumin (BSA) (Sigma) with subsequent Ficoll gradient centrifugation (densities: 1.108, 1.096, and 1.069). Islets, collected from the gradient, were then washed in HBSS supplemented with 0.3% BSA. Islet cells were gently dissociated by trypsin (Gibco), re-suspended in KRB solution (pH 7.4, BSA, 1.4 mM glucose and 0.5 mM EDTA), and filtered through a 70-μm cell strainer (Falcon). α- and β-cell populations were separated by flow cytometry fluorescence-activated cell sorting [FACS; Astrios Sorter (Beckman Coulter)], based on fluorescence intensity and wavelength, single cell nature, size, and viability. Purity of the sorted cells was further assessed by a second, additional FACS analysis. The viability of treated cells was evaluated by FACS with DRAQ7™ (Abcam) or DAPI (ThermoFisher) dyes.

### Immunohistochemistry

Sorted cells were plated on 35 mm dishes and fixed in 4% paraformaldehyde during 30 min. For immunohistochemistry, the fixed cells were incubated with mouse anti-Glucagon (1:500, Sigma) or guinea-pig anti-Insulin (1:500, ThermoFisher Scientific) primary antibodies. The signal was revealed by Alexa-Fluor anti-mouse 568 or Alexa-Fluor anti Guinea-pig 488 secondary antibodies, respectively (1:1,000, Molecular Probes). Bright-field and fluorescent images were obtained with EvosTL fluorescent microscope (ThermoFisher) using 4×, 10×, or 40× objectives.

### *In Vitro* Islets/Islet Cell Culture

For the *in vitro* culture, intact islets or sorted cells were recovered in RPMI 1640 complete medium (11.2 mM glucose, 110 μg/ml sodium pyruvate) supplemented with 10% fetal calf serum, 110 U/mL penicillin, 110 μg/ml streptomycin, and 50 μg/ml gentamycin and attached to 35 mm dishes or multi-well plates (LifeSystemDesign) pre-coated with a laminin-5-rich extracellular matrix (28). For hormone secretion assays, approximately 15,000 cells were plated per dish, in three separated drops of 50 μl each. For bioluminescence recordings either 250 islets or approximately 50,000 separated cells were plated per well.

### Quantitative RT-PCR (qRT-PCR)

Total RNA was prepared from homogenized islet cells using RNeasy^®^ Plus Micro Kit (Qiagen). Ten nanograms of total RNA were reverse transcribed (PrimeScript RT reagent kit; Takara) and pre-amplified (TaqMan PreAmp Master Mix; Applied Biosystems) following the manufacturer’s instructions. Specific target gene mRNA levels were analyzed by real-time quantitative PCR using the LightCycler technology (LC480; Roche Diagnostics). Mean values of gene expression were calculated from technical duplicates of each qRT-PCR analysis and normalized to the housekeeping gene *Hypoxanthine guanine phosphoribosyl transferase* (*Hprt*) exhibiting no significant variability of its expression level throughout each experiment, and therefore served as internal control. Primers used for this study are listed in Table 1.

**Table 1:** **List of primers used for quantitative RT-PCR**.

Target gene		Sequence primers, 5′-3′
Hprt	Forward	GCTCGAGATGTCATGAAGGAGAT
	Reverse	AAAGAACTTATAGCCCCCCTTGA
Clock	Forward	TTGCTCCACGGGAATCCTT
	Reverse	GGAGGGAAAGTGCTCTGTTGTAG
Per1	*Forward*	ACCAGCGTGTCATGATGACATAC
	*Reverse*	CTCTCCCGGTCTTGCTTCAG
Per2	*Forward*	GTAGCGCCGCTGCCG
	*Reverse*	GCGGTCACGTTTTCCACTATG
Ins1	*Forward*	TCTTCTACACACCCAAGT
	*Reverse*	TGCAGCACTGATCCACAA
Ins2	*Forward*	GCTCTCTACCTGGTGTGT
	*Reverse*	CTCCACCCAGCTCCAGTT
Gcg	*Forward*	GATCATTCCCAGCTTCCC
	*Reverse*	CTGGTAAAGGTCCCTTCA
MafA	*Forward*	CATTCTGGAGAGCGAGAA
	*Reverse*	TTTCTCCTTGTACAGGTC

### Hormone Secretion Measurements

Insulin and glucagon basal secretion assays were performed on approximately 15,000 attached separated α- or β-cells at 3 days *in vitro*. For basal insulin secretion assessment, cells were washed in KRB solution (Krebs-Ringer bicarbonate, pH 7.4, supplemented with 0.3% BSA) containing 2.8 mM glucose for 1 h, with subsequent incubation in KRB solution, containing 2.8 mM glucose for 30 min at 37°C in a cell culture incubator. For glucagon assessment, cells were washed in KRB solution containing 7 mM glucose for 1 h, followed by additional 30 min incubation in the same solution at 37°C in a cell culture incubator. To assess the islet cell acute secretory response, FACS-separated α- (*Venus*-positive) and β- (*Cherry*-positive) cells were subjected to 2 h incubation in KRB solution containing 5.6 mM glucose, followed by additional 30 min incubation in KRB containing 5.6 mM glucose (basal condition), 30 min incubation in KRB containing 5.6 mM glucose supplemented with 10 mM arginine (stimulated secretion for *Venus*-positive cells) or in KRB containing 16.7 mM glucose (stimulated secretion for *Cherry*-positive cells), and additional 30 min incubation in KRB containing 5.6 mM glucose (re-basal condition). At the end of each experiment, cells were lysed in Acid/Ethanol mixture (1.5% HCl/75% Ethanol) for glucagon or insulin residual contents measurements. Insulin and glucagon concentrations were quantified in the supernatants and in lysed cells by the Mouse Insulin or Glucagon ELISA kits (Mercodia). For acute secretion assays, glucagon and insulin values were expressed as a percentage of appropriate released hormone to residual cell content.

### Islet Cells Synchronization and Circadian Bioluminescence Monitoring

Adherent islets/islet cells were synchronized by a 1-h pulse of forskolin 10 μM (Sigma) prior to continuous bioluminescence recording in RPMI, supplemented with 100 μM luciferin (NanoLight Technology) (20). Photon counts of each well were integrated during 1 min, over 24 min intervals. For detrended time series, raw luminescence signals were smoothened by a moving average with a window of 24 h, allowing for a less biased comparison of bioluminescence values across experiments with regard to the measured circadian parameters (20).

## Results

### Separating Primary Mouse α- and β-Cells

**Figure 2:**
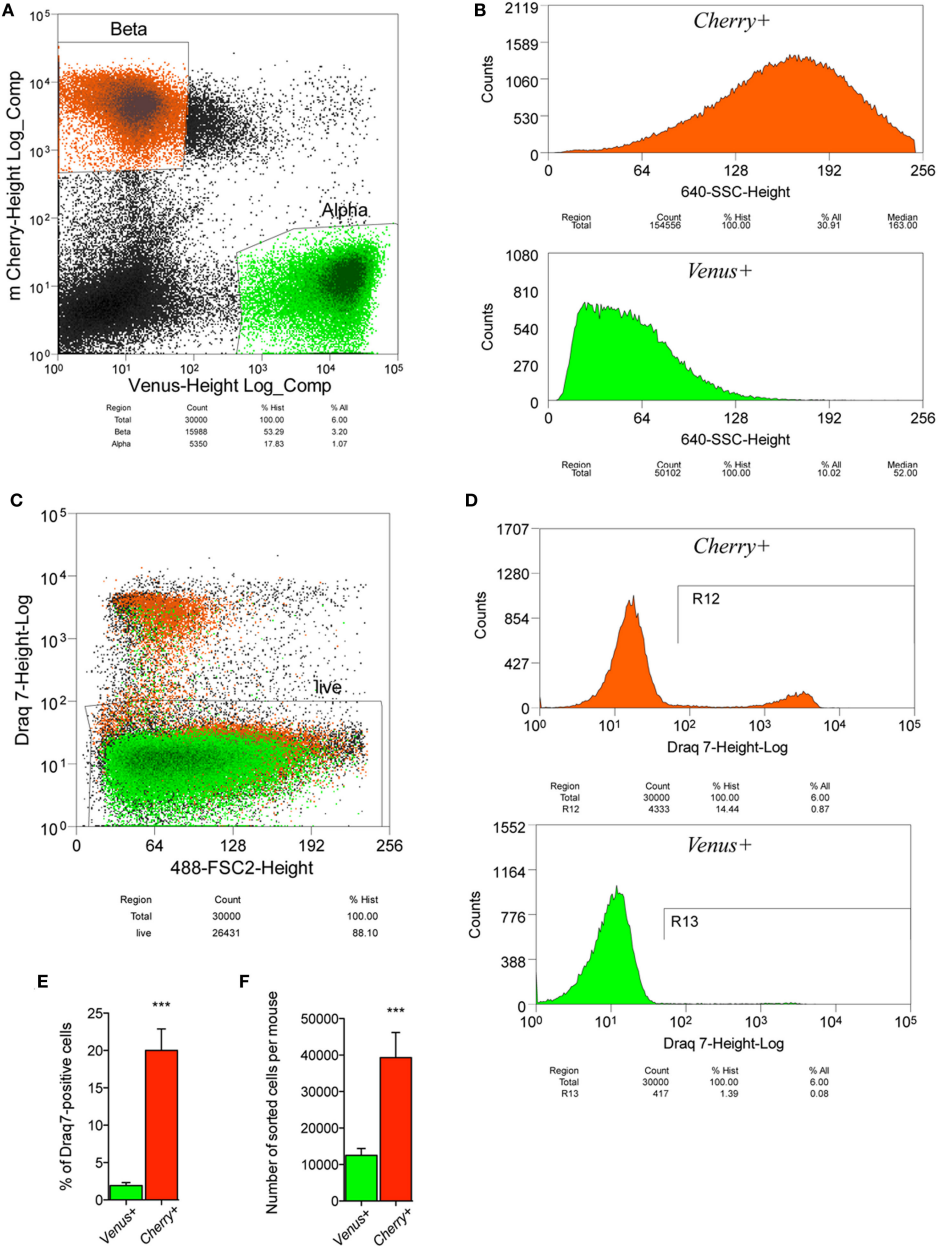
**Fluorescence-activated cell sorting of pancreatic islet cells from ProGcg-Venus/RIP-Cherry/Per2:Luc mice**. **(A)** Dot plots indicating good separation of *Venus*- and *Cherry*-positive cells from gated cell population. **(B)** Histograms representing number of analyzed events versus cell complexity (granularity) of the sorted cells. Note that *Cherry*-positive β-cells have greater granularity when compared to *Venus*-positive α-cells. **(C)** Dot plots for DRAQ7-based assessment of sorted cell viability, indicating approximately 90% alive cells in the preparation. **(D)** Representative histograms showing number of dead α- and β-cells among analyzed events and **(E)** corresponding quantitative data from 12 independent experiments (three to six mice per experiment), suggesting higher cell death for β-cell population, as compared to α-cells (paired, two-tailed Student’s *t*-test). **(F)** Histogram indicating average numbers of obtained α- and β-cells per mouse (*N* = 12 independent experiments with three to six mice per experiment), statistical difference illustrate more than three times greater content of β-cells within sorted population, as compared to α-cells (paired, two-tailed Student’s *t*-test). Data expressed as mean ± SEM.

**Figure 3:**
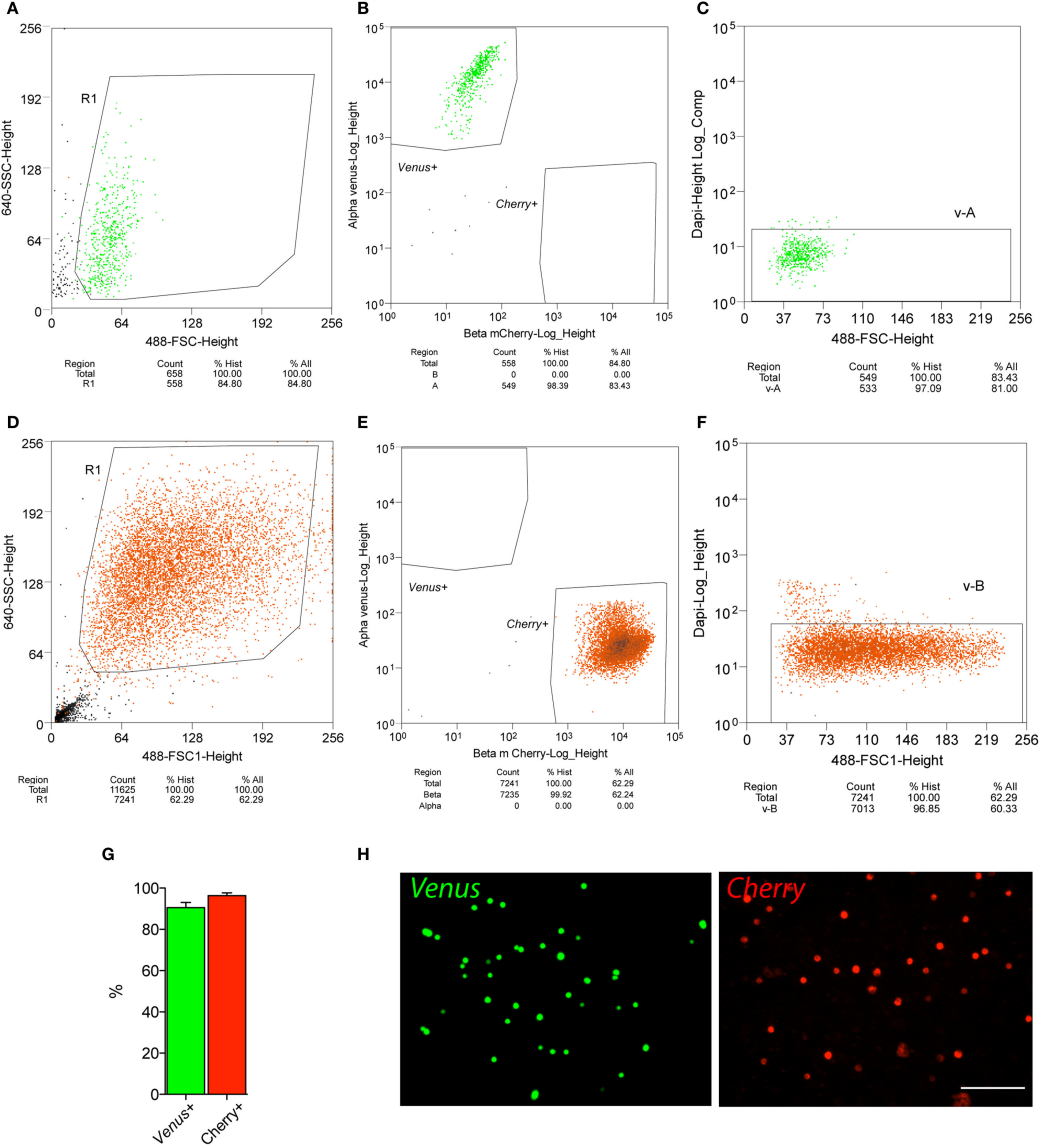
**Purity of α- and β-cell populations, separated by fluorescence-activated cell sorting (FACS) form ProGcg-Venus/RIP-Cherry/Per2:Luc mouse islets**. Sorted samples of *Venus*- and *Cherry*-positive cells were subsequently analyzed by the second run of FACS. **(A–C)** FACS-assessed purity analysis of obtained α-cell population: **(A)** dot plot presenting distribution of cell size [forward scatter detector (FSC), *x*-axis] versus granularity [side scatter detector (SSC), *y*-axis] of *Venus*-positive cells within gated cell population; **(B)** dot plot showing sample enrichment for *Venus*-positive cells and complete absence of *Cherry*-positive cells; **(C)** DAPI-assessed analysis of α-cell viability after sorting, indicating 97% viable cells in the sample. **(D–F)** FACS-assessed purity analysis of obtained β-cell population: **(D)** dot plot presenting distribution of cell size (FSC, *x*-axis) versus granularity (SSC, *y*-axis) of *Cherry*-positive cells within gated cell population; **(E)** dot plot showing enrichment of sample for *Cherry*-positive cells and absence of *Venus*-positive contaminants; **(F)** DAPI-assessed analysis of β-cell viability after sorting, indicating approximately 97% viable cells in the sample. **(G)** Histogram representing average purity in analyzed samples (data are presented as mean ± SEM, *N* = 6 experiments for α-cells; and *N* = 10 for β-cells). **(H)** Representative fluorescent images illustrating sorted *Venus*- and *Cherry*-positive cell populations (α- and β-cells, respectively). Scale bar = 100 μm.

In order to simultaneously label α- and β-cells within the islets, a *ProGcg*-*Venus*/RIP-*Cherry*/*Per2*:*Luc* reporter mouse line was established (Figure 1), allowing for the highly specific separation of nearly pure endocrine cell populations. To this end, following islet isolation and gentle trypsinization, dispersed cells were sorted by FACS based on cellular fluorescence characteristics, cell size (based on the data of Forward Scatter detector, FSC), and granularity (based on the data of Side Scatter detector, SSC; see Figures 2A,B). Of note, *Cherry*-positive cells showed higher cell granularity than *Venus*-positive cells (compare two histograms in Figure 2B). In parallel, cell viability was assessed by utilizing DRAQ7™, a dye that binds to DNA when cell membrane permeability is altered after initiation of cell death. Overall viability across preparations was near 90% (Figure 2C), with almost a 10-fold higher percentage of cell death for *Cherry*-positive cells (up to 20%) than for *Venus*-positive cells (Figures 2D,E), suggesting a higher sensitivity of *Cherry*-positive cells to the islet isolation, trypsinization, and/or sorting processes. The average number of harvested viable *Cherry*-positive cells per mouse was 39,292 ± 6,887, which is more than threefold higher than for *Venus*-positive cells (12,521 ± 1,885) (Figure 2F), reflecting the physiological ratio between these two cell types within mouse islets (22). In addition, the purity of the obtained *Venus*- and *Cherry*-positive cell populations was assessed by a complementary round of FACS analysis (Figures 3A–G). According to this analysis, the α-cell population comprises more than 90% viable *Venus*-positive cells without detectable contamination with *Cherry*-positive cells (Figures 3A–C,G), while the β-cell population contained up to 96% viable *Cherry*-positive cells without visible *Venus*-positive contaminants (Figures 3D,E,G). Finally, the morphological examination of sorted cell populations with a fluorescent microscope confirmed their high purity (Figure 3H).

### Primary Culture of Mouse α- and β-Cells

**Figure 4:**
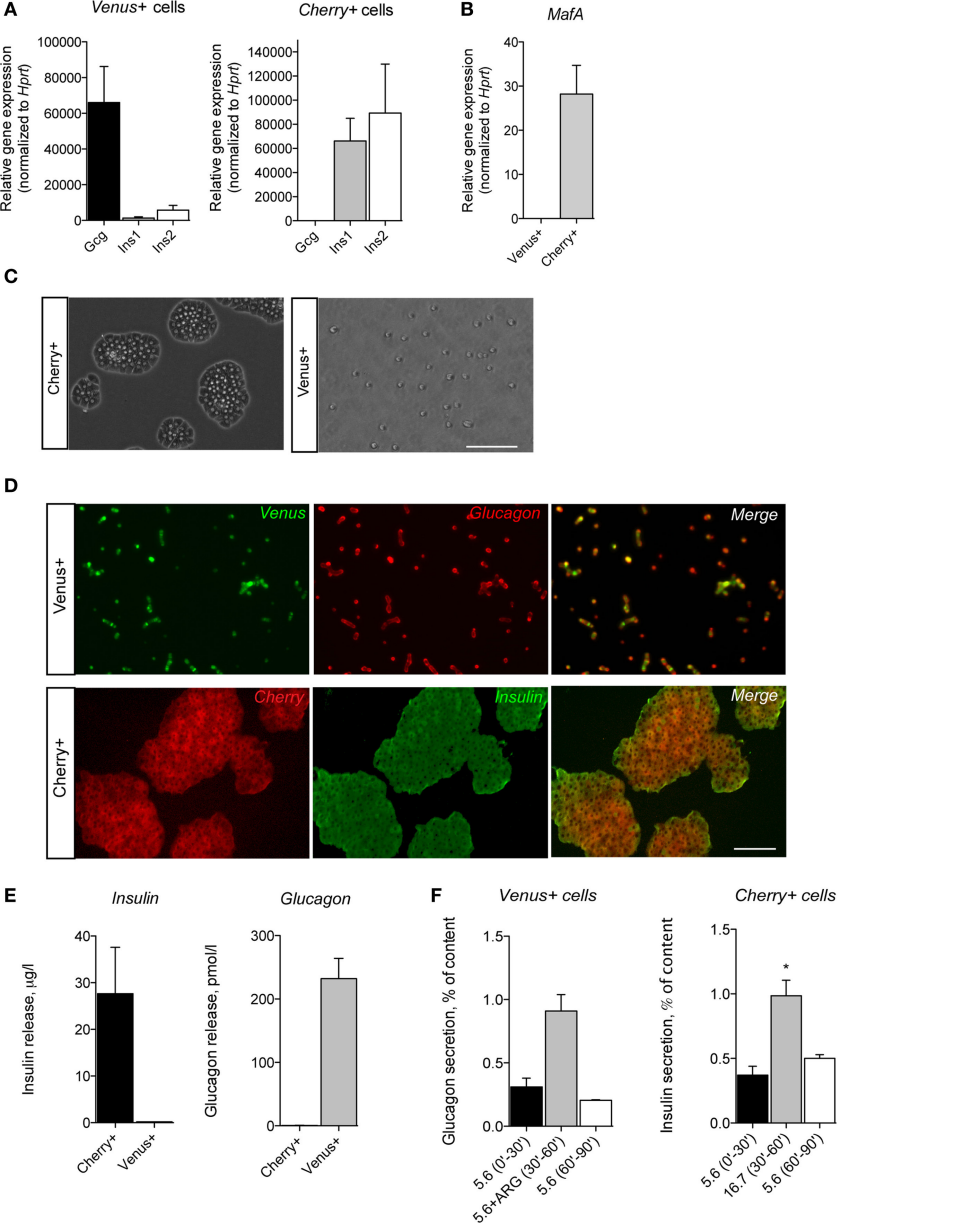
**Endocrine identity of sorted *Venus*- and *Cherry*-positive cell populations, isolated from ProGcg-Venus/RIP-Cherry/Per2:Luc mouse islets by fluorescence-activated cell sorting (FACS)**. **(A,B)** Quantitative RT-PCR analysis of **(A)** insulin (*Ins1* and *Isn2*) and glucagon (*Gcg*) transcripts; or **(B)** V-Maf avian musculoaponeurotic fibrosarcoma oncogene homolog A (*MafA*) β-cell-specific transcript in freshly isolated *Venus*- and *Cherry*-positive cells. Data are presented as relative expression of the target gene normalized to the *Hprt* housekeeping gene (mean ± SEM, *N* = 3 independent experiments for **(A)** and *N* = 4 experiments for **(B)** with six mice each). **(C)** Representative bright-field images of cultured *Venus-* and *Cherry*-positive cells 48 h after plating. **(D)** Representative fluorescent images of ProGcg-Venus and Insulin-Cherry transgenic proteins (left panels) colocalizing with glucagon and insulin immunostaining, respectively (merge, right panels). Analysis of purified *Venus-* and *Cherry*-positive cell cultures prior the immunostaining showed no cross-contaminations. No *Venus*-positive cells appeared positive for insulin, and no *Cherry*-positive cells appeared positive for glucagon by immunostaining. Scale bars = 100 μm. **(E)** FACS-separated *Venus*- and *Cherry*-positive cells were plated in 35 mm dishes (approximately 15,000 cells per dish, as described in Section “Material and Methods”), and subjected to 30 min incubation with 2.8 mM glucose (for basal insulin assessment) or with 7 mM glucose (for basal glucagon assessment). Hormone levels were assessed in the incubation solution with Mouse Insulin and Glucagon ELISA kits (Mercodia). Data are expressed as absolute values (mean ± SD) for *N* = 6 mice used for two independent islet preparations. **(F)** Acute secretion assays performed on cultured *Venus*- and *Cherry*-positive cells 48 h after plating. Cell supernatants were collected for three successive 30-min periods (30 min basal condition, 30 min stimulated condition, and 30 min re-basal condition, as described in Section “Material and Methods”) after 2 h of cell depletion in KRB solution. Hormone levels were assessed with Mouse Insulin and Glucagon ELISA kits and normalized to the residual hormone content in the end of the experiment. Data are expressed as percentage of glucagon or insulin from cell contents (mean ± SD; *N* = 2 experiments for *Venus-*positive cells and *N* = 4 experiments for *Cherry*-positive cells (two mice per experiment); * *p* < 0.05, two-tailed Student’s *t*-test).

Insulin and glucagon transcript expression levels were assessed in separated *Venus*- and *Cherry*-positive cells by qRT-PCR analysis. As expected, *Gcg* transcription was the highest in the *Venus*-positive population, further confirming the α-cell identity, while both insulin transcripts *Ins1* and *Ins2* were abundant in *Cherry*-positive cells, indicating their β-cell identity (Figure 4A). Importantly, expression levels of the opposite cell hormone genes (*Ins1* and *Ins2* in *Venus*-positive cells, and *Gcg* in *Cherry*-positive cells) were more than 10-fold (for *Ins1* and *Ins2*) and 1,000-fold (for *Gcg*) lower, further confirming the satisfactory purity of the α- and β-cell populations. Furthermore, *Cherry*-positive cell population expressed β-cell-specific transcription factor *MafA* at the levels which were 1,000-fold higher compared to this transcript expression in *Venus*-positive counterparts (Figure 4B).

For cell culture, separated α- and β-cells were plated on plastic dishes covered with laminin-enriched matrix, which improves islet cell survival and function, and keeping them from de-diffentiation (28). Attached islet cells were maintained *in vitro* for at least 10 days. During the first 48 h of culture, attached β-cells formed monolayer cell aggregates resembling pseudo islets, while α- cells formed small domed structures composed by a few cells or stayed separated (Figure 4C). Immunofluorescence analysis demonstrated that *Venus*-positive cells in culture co-localized with glucagon-specific antibody, whereas *Cherry*-positive cells co-localized with insulin-specific antibody (Figure 4D), further validating these cell identity and high purity of α- and β-cell fractions.

Hormone secretion assays, performed after 3 days in culture, detected high basal levels of insulin in the supernatant of β-cells, and high basal levels of glucagon in α-cell supernatants, released during 30 min in the presence of constant glucose concentrations (2.8 mM for insulin and 7 mM for glucagon; Figure 4E). Noteworthy, secretion of the opposite hormones (glucagon by β-cells and insulin by α-cells) was below the detection level. Importantly, incubation of cultured α-cells with arginine induced about 2.5-fold increase in glucagon secretion, whereas incubation of cultured β-cells with high glucose stimulated secretion of insulin above two-fold (Figure 4F). Taken together, these data suggest that the here-described methodology ensures highly specific and efficient separation of the two main populations of islet cells, resulting in nearly pure populations of viable and functional α- and β-cells, which can be maintained in culture.

### Assessment of Circadian Oscillator Properties in Separated α- and β-Cells

**Figure 5:**
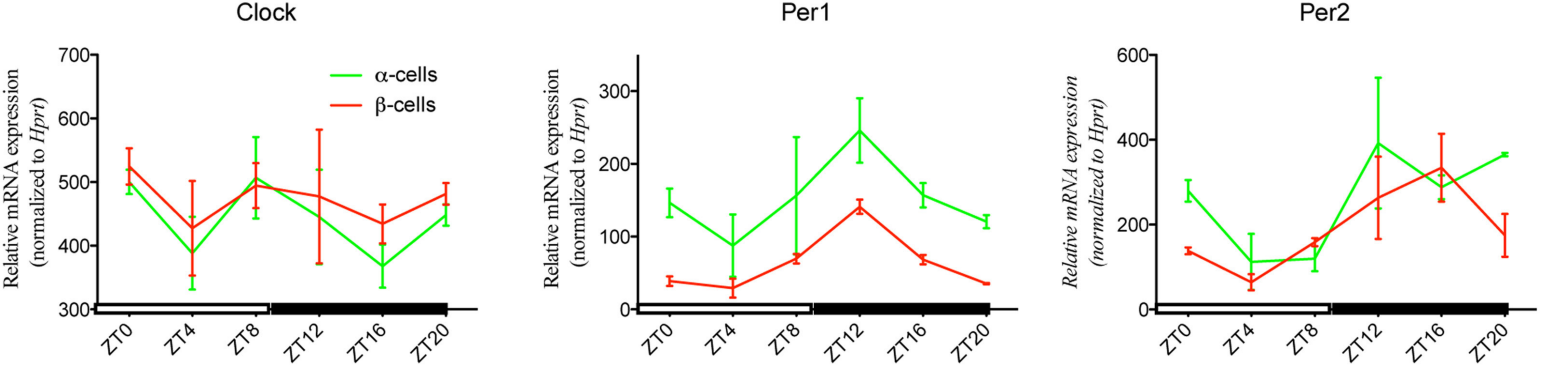
**Isolated around-the-clock α- and β-cells express *in vivo* circadian rhythm for selected core clock genes after fluorescence-activated cell sorting (FACS) separation**. Triple transgenic mice (ProGcg-Venus/RIP-Cherry/Per2:Luc) were kept at standard 12 h/12 h light–dark cycles (ZT0 (Zeitgeber) corresponds to 07:00 a.m., and ZT12 corresponds to 07:00 p.m.), with constant access to the drinking water and night-restricted feeding regime for 2 weeks prior to islet collection. Pancreatic islets were isolated around-the-clock every 4 h and subjected to the FACS separation procedure. Temporal profiles of selected core clock transcripts (*Clock, Per1*, and *Per2*) were assessed in freshly extracted mRNA at each time point from α- and β-cell populations by quantitative RT-PCR analysis. Transcript levels were normalized to *Hprt* expression. Data expressed as mean ± SD (*N* = 2 biological replicates of 6 mice at each time point).

In view of the complexity of the separation procedure by FACS, we next explored if separated α- and β-cell populations maintain their circadian properties, as was previously reported to be the case in isolated intact islets (11, 18). To this end, mRNA levels for selected core clock transcripts have been assessed in sorted islet cells isolated every 4 h during 24 h. The qRT-PCR analysis revealed pronounced rhythmic patterns for *Per1* and *Per2* genes over 24 h, while the oscillation of *Clock* transcription was shallow, in agreement with previous reports (Figure 5) (11, 19).

**Figure 6:**
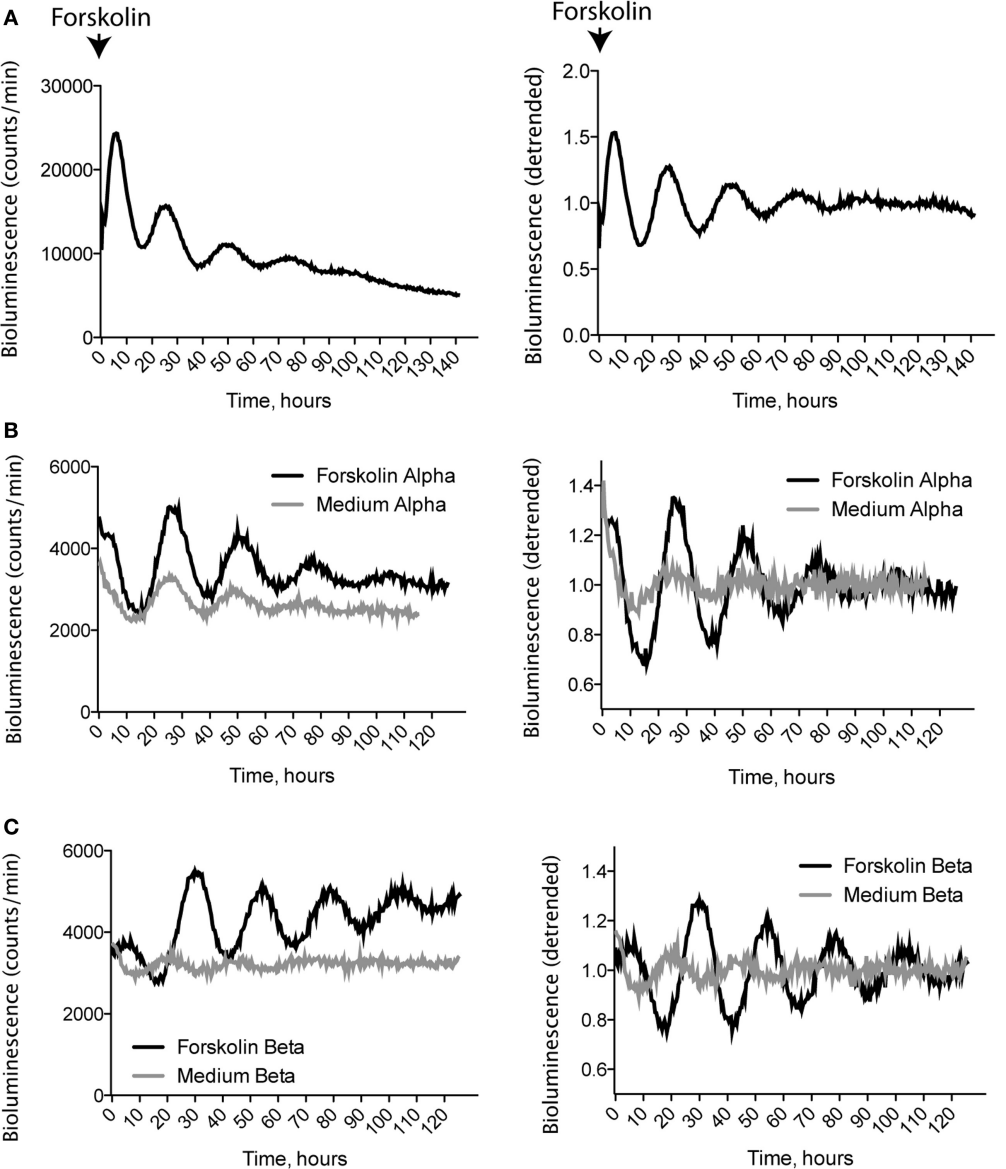
***In vitro* monitoring of circadian rhythm in fluorescence-activated cell sorting-separated α- and β-cells**. Representative Period2:Luciferase oscillation profiles of cultured whole islets **(A)**, α- cells **(B)**, and β-cells **(C)** isolated from ProGcg-Venus/RIP-Cherry/Per2:Luc mice, following 1-h synchronization with 10 μM forskolin or fresh medium **(A,B)**. Data are presented as absolute (left panels) or corresponding detrended (19) values (right panels).

Moreover, we explored cell-autonomous molecular clocks in separated α- and β-cell primary cultures synchronized *in vitro*. Similar to intact islets (Figure 6A), populations of pure α-cells (Figure 6B) and β-cells (Figure 6C) responded to a 1 h synchronizing pulse of forskolin by demonstrating high-amplitude self-sustained circadian oscillations of *Per2:Luc* reporter expression for at least 5 consecutive days following synchronization. Synchronizing effect of forskolin on cultured α- and β-cells was specific, since medium change alone had little synchronizing effect on the *Per2:Luc* expression in both cell types (Figures 6B,C). Collectively, these data suggest that circadian oscillations persist in pure populations of α- and β-cells following islet isolation, trypsinization, and FACS separation procedures.

## Discussion

The major obstacle for studying α- and β-cells is that they are organized in the tight three-dimensional structure within the pancreatic islet. We successfully overcame this problem by utilizing transgenic mice, specifically expressing the *ProGcg*-*Venus* reporter in α-cells (23) and the RIP-*Cherry* reporter in β-cells (24) (Figure 1), allowing to separate these two cell populations by FACS with high viability and purity (Figures 2 and 3). The here-described methodology allows for extracting up to 40,000 β-cells and 12,500 α-cells per mouse (Figure 2F), and culturing thus separated primary α- and β-cells for at least 10 days. Importantly, the cell ratio after sorting reflects proportion between α- and β-cells in mouse pancreatic islets *in vivo* (60–80 versus 15–20%, respectively) (22, 29). The value obtained by FACS for this islet cell ratio following isolation and separation provides an estimation for the islet cell composition, which gives an advantage for studying the altered ratio between α- and β-cells upon different pathological conditions like obesity, T2D and others.

In an agreement with the previous studies, we demonstrate that a reporter-based separation of endocrine cells from pancreatic islets allows to obtain α- and β-cell populations bearing high expression levels of *glucagon*, and *insulin* and *MafA* transcripts, respectively (Figures 4A,B) (30, 31). Importantly, recently published by us circadian RNA sequencing analysis of thus separated α- and β-cell populations provides further extensive characterization of their differential transcriptional patterns (32). Indeed, *insulin, glucagon, MafA, Arx*, and additional cell-specific transcripts were expressed in a highly specific manner in the appropriate islet cell type (32). Additionally, separated primary α- and β-cells exhibited cell-specific glucagon and insulin positive immunostaining, respectively (Figure 4D), and inherent basal hormone release properties (Figure 4E). Moreover, cultured islet cells responded properly to the physiologically relevant secretagogues (high glucose for insulin, and arginine for glucagon), by inducing the respective hormone secretion about twofolds (Figure 4F). In line with these data, our recent work demonstrated that primary α-cells isolated from *ProGcg-Venus* mice responded to high glucose by a reduction in glucagon release (33), thus giving the opportunity to perform hormone secretion studies by these cells *in vitro*. This is particularly important for α-cells, since in mixed islet cell populations the glucagon secretion is altered by the amount of insulin secreted by adjacent β-cells, which represent the cell majority (34, 35). Furthermore, we have recently shown that both basal insulin secretion by synchronized β-cells and basal glucagon secretion by synchronized α-cells are circadian, further validating that thus isolated islet cells keep their cell-autonomous clocks and their functional properties (32).

Functional circadian oscillators have been previously characterized in mouse pancreatic islets (11, 18, 21, 36). However, circadian studies conducted in whole islets principally assess the more abundant β-cell population, and are unable to exclude complex functional interactions between different endocrine cell types (34, 35). At the same time, the circadian characteristics of α-cells remain largely unexplored and have only been assessed in a single study in primary cells to the best of our knowledge (37). The here presented efficient separation of islet cell populations paves the way for systematic analyses of circadian transcriptional outputs of the clock in pure populations of α- and β-cells *in vivo* (Figure 5), and *in vitro* following different synchronization stimuli upon selected conditions (as exemplified by forskolin synchronization in Figure 6). Our *in vivo* studies revealed circadian oscillations of *Per1* and *Per2* transcripts, exhibiting peak expression levels in the beginning of the dark phase in α- and β-cells, in accordance with a previous report for the intact islets (11). In contrast, the temporal pattern of *Clock* expression was shallow, in agreement with earlier studies in mouse liver *in vivo* (26) and in synchronized human islets *in vitro* (19). These data strongly suggest that circadian oscillators persist in isolated α- and β-cells, following not only islet isolation procedure as previously demonstrated by Marcheva et al. (11), but also further trypsinization and FACS separation. Finally, in agreement with our previous observations in dispersed human islet cells compared to intact human islets (19, 20), our results further support that the three-dimensional islet structure is not essential for maintaining cell-autonomous molecular clocks in α- and β-cells (Figure 6). In agreement with previous publications (11, 18), demonstrating strong *in vitro* synchronizing properties of forskolin in pancreatic islets, we show high-amplitude, self-sustained circadian oscillations induced by a forskolin pulse in isolated islets, and in separated α- and β-cells (Figure 6). Moreover, our recent study suggests that α- and β-cell oscillators possess distinct circadian properties *in vivo* and *in vitro* (32). Importantly, this methodology allows to study the cell-autonomous impact of functional clocks on α- and β-cell hormone secretion *in vitro*, for instance by islet cell perifusion, as reported by us (38). The here-described strategy to study the circadian oscillator in separated primary mouse α- and β-cells will help to unravel the important functional roles of these cells in the regulation of glucose metabolism under physiological conditions, and upon metabolic diseases, including obesity and T2D.

## Ethics Statement

Animal studies were reviewed and approved by the Veterinary Office of the State of Geneva (authorization numbers No 1028/3919/1 and GE/159/15).

## Author Contributions

VP contributed to data acquisition, analysis and interpretation, and drafted the manuscript. YG contributed to data acquisition and analysis. CD designed the study, contributed to the data acquisition and analysis, and drafted the manuscript. All authors took part in the revision of the manuscript and approved the final version.

## Conflict of Interest Statement

The authors declare that the research was conducted in the absence of any commercial or financial relationships that could be construed as a potential conflict of interest.
